# Does implementation of a hospitalist program in a Canadian community hospital improve measures of quality of care and utilization? an observational comparative analysis of hospitalists vs. traditional care providers

**DOI:** 10.1186/1472-6963-13-204

**Published:** 2013-06-05

**Authors:** Vandad Yousefi, Christopher AKY Chong

**Affiliations:** 1 Department of Medicine, Lakeridge Health, Oshawa, ON, Canada; 2 Department of Family Practice, Vancouver General Hospital, Vancouver, BC, Canada; 3 Faculty of Health Sciences, Queen’s University, Kingston, ON, Canada

## Abstract

**Background:**

Despite the growth of hospitalist programs in Canada, little is known about their effectiveness for improving quality of care and use of scarce healthcare resources. The objective of this study is to compare measures of cost and quality of care (in-hospital mortality, 30-day same-facility readmission, and length of stay) of hospitalists vs. traditional physician providers in a large Canadian community hospital setting.

**Methods:**

We performed a retrospective analysis of data from the Canadian Institute for Health Information (CIHI) Discharge Abstract Database, using multivariate logistic and linear regression analyses comparing performance of four provider groups of traditional family physicians (FPs), traditional internal medicine subspecialists (other-IM), family physician-trained hospitalists (FP-Hospitalist), and general internal medicine-trained hospitalists (GIM-Hospitalist).

**Results:**

Compared to traditional FPs, FP-Hospitalists and GIM-Hospitalists demonstrate lower mortality [OR 0.881, (CI 0.779 – 0.996); and OR 0.355, (CI 0.288 – 0.436)] and readmission rates [OR 0.766, (CI 0.678 – 0.867); and OR 0.800, (CI 0.675 – 0.948)]. Compared to traditional FPs, GIM-Hospitalists appear to improve length of stay [OR−2.975, (CI −3.302 – -2.647)] while FP-Hospitalists perform similarly [OR 0.096, (CI −0.136 – 0.329)]. Compared to other-IM, GIM-Hospitalists have similar performance on all measures while FP-Hospitalists show a mixed impact.

**Conclusions:**

Compared to traditional family physicians, hospitalists appear to improve measures of quality and resource utilization. Specifically, hospitalists demonstrate lower in-hospital mortality and 30-day readmission rates while improving (or at least showing similar) length of stay. Compared to traditional subspecialists, hospitalists demonstrate similar performance despite looking after sicker and more complex medical patients.

## Background

Since hospitalist programs first emerged over two decades ago, their numbers have grown rapidly in North America. At many healthcare institutions, hospitalists have increasingly replaced primary care providers and subspecialists as the main providers of general medical inpatient care [1].

A number of general medical care models have traditionally existed in Canadian hospitals [2]. In academic centres, general internists have essentially worked as hospitalists as part of clinical teaching units (CTUs). In larger urban and semi-urban community hospitals, most medical patients have had their own primary care giver (family physician or general practitioner) act as their Most Responsible Physician (MRP), with some being directly admitted to the care of medical subspecialists. In rural hospitals, family physicians have almost exclusively provided care to hospitalized patients.

The 1990s witnessed the first emergence of Canadian hospitalist programs as increasing numbers of primary care givers began surrendering their admitting privileges due to workload pressures and inadequate remuneration [2,3]. At the same time, more narrowly defined subspecialists shifted away from the MRP role as hospitalized patients became increasingly complex [2]. In response to a growing crisis in inpatient physician coverage, healthcare institutions developed hospitalist programs staffed by “generalist” physicians [4]. While some earlier work indicated that the majority of Canadian hospitalists are trained as family physicians, an increasing number of general internists are also entering the field [2,5,6].

Currently, the majority of research on the cost effectiveness and quality of care delivered by hospitalists has come from programs in the United States [7,8]. Most studies suggest that compared to traditional models, hospitalist care results in lower length of stay, reduced costs and higher quality of care for select populations. Proponents of the model argue that hospitalists can better maintain diagnostic and procedural skills due to higher inpatient exposure and the “practice makes perfect” concept [9,10]. They also propose that hospital-based physicians develop a better understanding of institutional processes, which in turn results in better efficiency and resource utilization. However, many of these studies have suboptimal methodological qualities and have not adequately controlled for confounding clinical factors [8]. It is also unclear if such findings can be extrapolated to other hospitals operating under fundamentally different national health care systems such as those in Canada.

Over the past 10 years, our institution has gradually implemented a hospitalist program staffed by both family physicians and general internists. The purpose of this study is to compare quality of care and resource utilization of hospitalists compared to traditional care providers in a Canadian hospital. Moreover, we aim to explore potential differences between hospitalists with different training backgrounds.

## Methods

Lakeridge Health (LH) is a large community hospital network in southeastern Ontario comprised of four campuses that together provide a wide range of services. In 2001, a hospitalist program was initiated at the largest campus (LH Oshawa) with five family physicians recruited from the local community (“FP-Hospitalists”). This program has expanded to 16 full time equivalent family physician-trained hospitalists who care for acute medical, oncology, renal-dialysis, stroke, palliative and rehab inpatients. FP-Hospitalists also provide medical consults to surgeons, co-manage patients with the orthopedic and psychiatry programs and provide care to healthy newborns. In 2005, a sister hospitalist program was instituted at the Oshawa campus, comprised of 2 general internists (“GIM-Hospitalists”). This program has since expanded to include 4 full-time equivalent GIM-Hospitalists who provide care to a similar population as the FP-Hospitalists although with a heavier emphasis on cardiac patients. The general internists also provide coverage for the stroke prevention and pre-operative clinics, as well as medical consultations to subspecialists and FP-Hospitalists. Almost all FP-Hospitalists spend 100 percent of their clinical time delivering inpatient care, while the GIM-Hospitalists spend 25 percent of their time covering the above-mentioned hospital-based ambulatory clinics.

While the FP- and GIM -Hospitalists are funded and administrated separately, there is a close working relationship between the two groups. For example, GIM-Hospitalists admit all medical patients from the Emergency Department (ED) during the day, while FP-Hospitalists admit patients in the evenings. Both groups are geographically distributed, such that FP-Hospitalists attend to patients on three acute medical units while the GIM-Hospitalists are responsible for one medical ward that includes telemetry beds. As a result, patients admitted by one group may be looked after by the other. Both groups attend patients on the Short Stay Unit and patients boarded in the ED. Members of both groups also participate in code blue and code stroke coverage, ICU transfers to the ward, and various quality improvement initiatives. The FP-Hospitalists work 13 weeks in a 16-week rotation, while GIM-Hospitalists work 3 weeks out of 4.

A declining number of community family physicians (“traditional FPs”) continue to admit and look after their own patients at the Oshawa campus. As well, some inpatients with clearly defined medical problems continue to be cared for by internal medicine subspecialists (“other-IM”), particularly cardiologists and gastroenterologists.

In order to study care differences among our institution’s two traditional physician staffing models (traditional FPs and other-IM) and newer hospitalist programs (FP-Hospitalist and GIM-Hospitalist), we used a national administrative database maintained by the Canadian Institute of Health Information (CIHI). Canadian hospitals provide a large number of demographic, diagnostic and outcomes data on all hospitalization episodes to the CIHI Discharge Abstract Database (DAD). The DAD uses the International Classification of Diseases and Canadian Classification of Health Interventions systems to categorize and code diagnoses and therapeutic interventions. CIHI has also devised a Case Mix Grouping (CMG) methodology to group patients with similar resource utilization under the same diagnostic categories. In 2007, CIHI introduced an enhanced methodology (called CMG Plus) that replaced the earlier CMG groupings. Moreover, CIHI has developed a methodology for measurement of Hospital Standardized Mortality Ratios (HSMR) which is reported nationally.

Through the Decision Support department of LH, we obtained DAD data on all adult admissions to LH Oshawa from April 2003 through to March 2010. Inclusion criteria were all patients 18 years and older admitted to the medical program. We excluded surgical, obstetrical and psychiatric cases, and cases for which the primary care providers were non-medical (refer to Appendix A for details). The main quality outcome measures were in-hospital mortality and 30-day same-facility readmission rate, and length of stay (LOS) was used as a surrogate for resource utilization. We conducted multivariate logistic and linear regression analyses to identify demographic and clinical factors affecting these outcome measures, including physician provider groups (refer to Appendix A for statistical details). We also examined HSMRs calculated using the CIHI methodology, with 95% confidence intervals calculated using Byar’s approximation [11]. Data were analyzed using SPSS version 19. The Research Ethics Board of Lakeridge Health approved the study.

## Results

From April 2003 to March 2010, 124 554 patients were admitted to LH Oshawa, of which 34 524 patients met inclusion criteria. Table 1 provides the baseline characteristics of patients admitted to the four provider groups over three time periods. The results indicate a gradual growth in the number of patients admitted to FP- and GIM- Hospitalists, with a concomitant decrease in the number of patients cared for by traditional providers (other-IM and traditional FP groups). It also demonstrates that over time, the hospitalist groups (particularly FP-Hospitalist) cared for sicker and more complex patients, as evidenced by higher Charlson comorbidity scores and higher number of interventions.

**Table 1:** Baseline characteristics of patients admitted Lakeridge Health Oshawa, April 2003 to March 2010

	**April 2003 – March 2006**	**April 2006 to March 2008**	**April 2008 to March 2010**
	**Total N = 14 993**	**Total N = 9521**	**Total N = 10010**
**n (% of Total N)**			
Traditional-FP	2190 (14.7%)	429 (4.5%)	292 (2.9%)
FP-Hospitalist	6044 (40.3%)	5164 (54.2%)	5267 (52.6%)
GIM-Hospitalist	42 (0.3%)	498 (5.2%)	1595 (15.9%)
other IM	6717 (44.8%)	3430 (36.0%)	2856 (28.5%)
**n female (% of cases within provider group)**			
Traditional-FP	1241 (56.7%)	238 (55.5%)	135 (46.2%)
FP-Hospitalist	3302 (54.6%)	2825 (54.7%)	2843 (54.0%)
GIM-Hospitalist	20 (47.6%)	274 (55.0%)	814 (51.0%)
Other IM	3101 (46.2%)	1541 (44.9%)	1198 (41.9%)
**Mean age in years (SD)**			
Traditional-FP	68.09 (19.23)	69.28 (17.95)	69.44 (15.53)
FP-Hospitalist	68.54 (17.28)	69.98 (16.83)	69.89 (16.97)
GIM-Hospitalist	64.95 (18.54)	66.24 (19.37)	68.84 (17.54)
Other IM	63.51 (16.83)	63.01 (16.69)	62.84 (16.44)
**n Charlson score ≥ 3 (% of cases within provider group)**			
Traditional-FP	583 (26.6%)	173 (40.3%)	116 (39.7%)
FP-Hospitalist	2052 (34.0%)	2448 (47.4%)	2656 (50.4%)
GIM-Hospitalist	10 (23.8%)	194 (39.0%)	644 (40.4%)
Other IM	1389 (20.7%)	941 (27.4%)	868 (30.4%)
**n Interventions (SD)**			
Traditional-FP	0.51 (1.00)	0.51 (0.94)	0.61 (1.07)
FP-Hospitalist	0.63 (1.10)	0.76 (1.22)	0.83 (1.25)
GIM-Hospitalist	0.17 (0.66)	0.44 (0.86)	0.57(1.07)
Other IM	0.54 (0.85)	0.69 (1.00)	0.84 (1.07)

Legend: FP = family physician, GIM = general internal medicine, IM = internal medicine, SD = standard deviation.

Table 2 outlines the top five CMGs for the various physician groups over the study period. There are overlaps in the most common CMGs for traditional FPs, FP-Hospitalist, and GIM-Hospitalist groups (particularly for chronic obstructive lung disease, pneumonia and heart failure). However, differences do exist, with a more enriched palliative population in the traditional FP and FP-Hospitalist groups. As well, the other-IM group is clearly dominated by admissions to cardiologists. It is important to note that the time periods presented in Table 2 differ from Table 1. This is due to the introduction of the CMG Plus methodology in 2007 that replaced the earlier version.

**Table 2:** Top five Case Mix Groups*

	**Top 5 CMG Plus, from 2007 to 2010 (n)**	**Top 5 CMG, from 2003 to 2007 (n)**
**Traditional-FP**	- Palliative care (36)	- Simple pneumonia and pleurisy (184)
**N = 2911**	- COPD (31)	- Specific cerebrovascular disorders except transient is chemic attacks (178)
	-Viral/unspecified pneumonia (28)	- Esophagitis, gastroenteritis and miscellaneous digestive disease (140)
	- General symptoms/signs (26)	- COPD (114)
	- Heart failure without cardiac catheter (23)	- Chronic bronchitis (106)
**FP-Hospitalist**	- COPD (660)	- COPD (470)
**N = 16 475**	- Palliative care (455)	- Chronic bronchitis (434)
	- Viral/unspecified pneumonia (370)	- Other specified aftercare (405)
	- Heart failure without cardiac catheter (352)	- Esophagitis, gastroenteritis and miscellaneous digestive disease (404)
	- Ischemic event of central nervous system (312)	- Heart failure (401)
**GIM-Hospitalist**	- COPD (237)	- AMI without cardiac catheter without specified cardiac conditions (12)
**N = 2135**	- Heart failure without cardiac catheter (221)	- Arrhythmia (8)
	- Viral/unspecified pneumonia (89)	- Heart failure (7)
	- Diabetes (77)	- Diabetes (6)
	- Myocardial infarction/shock/arrest without cardiac catheter (77)	- Chest pain (6)
**Other IM**	- Myocardial infarction/shock/arrest with cardiac catheter (498)	- AMI without cardiac catheter without specified cardiac conditions (757)
**N = 13 002**	- Arrhythmia without cardiac catheter (306)	- Arrhythmia (584)
	- Angina (except unstable)/chest pain without cardiac catheter (271)	- Heart failure (559)
	- Unstable angina/atherosclerotic heart disease without cardiac catheter (205)	- Unstable angina without cardiac catheter without specified cardiac conditions (531)
	- Heart failure without cardiac catheter (204)	


Legend: *Case Mix Groups (CMG) are aggregates of acute care inpatients with similar resource utilization; the Canadian Institute of Health Information developed CMG for use until 2007 when an enhanced version (CMG+) was introduced.

FP = family physician, GIM = general internal medicine, IM = internal medicine, COPD = chronic obstructive pulmonary disease, AMI = acute myocardial infarction.

Table 3 provides unadjusted mortality, readmission and LOS for our study groups, while Table 4 illustrates the results of multivariate analysis for the main outcome measures after controlling for significant clinical and demographic factors. Compared to traditional FPs, both FP- and GIM-Hospitalists have statistically significant lower adjusted in-hospital mortality rates (p = 0.043 and p < 0.001 respectively), with a greater magnitude of effect observed for the GIM-Hospitalist group. This finding is also reflected in the HSMRs (Table 5). However, results of comparisons to other-IM are mixed. Compared to other-IM, the GIM-Hospitalist group has a similar adjusted-mortality rate in logistic regression, but CIHI calculated HSMRs are much lower. On the other hand, FP-Hospitalists appear to have a higher mortality rate than other-IM, but CIHI calculated HSMRs are similar. Adjusted 30-day readmission rates of the FP-Hospitalist, GIM-Hospitalist and other-IM groups all appear similarly lower than the traditional FP group. Finally, while FP-Hospitalist LOS was similar to traditional FPs, GIM-Hospitalists have a statistically lower adjusted LOS by about three days. The other-IM group appears to perform similarly in LOS to the GIM-Hospitalist group.

**Table 3:** Crude hospital mortality, 30-day readmission, lengths of stay for study groups

	**Traditional-FP**	**FP-Hospitalist**	**GIM-Hospitalist**	**Other IM**
	**N = 2911**	**N = 16 475**	**N = 2135**	**N = 13 002**
**% death (n)**				
	13.2% (383)	14.0% (2310)	7.0% (149)	6.0% (779)
**% readmitted (n)**	12.9% (376)	11.0% (1806)	13.2% (283)	12.6% (1644)
**mean LOS in days (SD)**	8.90 (12.58)	9.97 (12.19)	5.64 (7.88)	4.26 (4.30)

LOS = length of stay, SD = standard deviation, FP = family physician, GIM = general internal medicine, IM = internal medicine.

**Table 4:** Multivariate regressions for predictors of hospital mortality, length of stay and 30-day readmission

**Model**	**Clinical variable**	**Odds ratio (95% CI)**	**p value**
Logistic regression for hospital mortality (n =34523)	Provider Group		
	Traditional-FP	Reference	
	FP-Hospitalist	0.881 (0.779 – 0.996)	0.043
	GIM-Hospitalist	0.355 (0.288 – 0.436)	<0.001
	Other-IM	0.490 (0.426 – 0.562)	<0.001
	Age	1.025 (1.022 – 1.028)	<0.001
	Charlson score		
	0	Reference	
	1–2	3.300 (2.816 – 3.868)	<0.001
	3 or more	9.417 (8.072 – 10.988)	<0.001
	Length of stay		
	0 – 1 days	2.847 (2.563 – 3.161)	<0.001
	2 – 9 days	Reference	
	10 or more days	1.282 (1.174 – 1.399)	<0.001
	Number of interventions		
	0	Reference	
	1	0.909 (0.829 – 0.997)	0.044
	2 or more	1.278 (1.154 – 1.414)	<0.001
	**Clinical variable**	**Odds ratio (95**% **CI)**	**p value**
Logistic regression for 30-day readmission (n = 30902)	Provider Group		
	Traditional-FP	Reference	
	FP-Hospitalist	0.766 (0.678 – 0.867)	0.010
	GIM-Hospitalist	0.800 (0.675 – 0.948)	0.004
	Other-IM	0.831 (0.734 – 0.941)	<0.001
	Age	1.005 (1.003 – 1.007)	<0.001
	Initial discharge to long-term care facility	0.443 (0.393 – 0.500)	<0.001
	Charlson score		
	0	Reference	
	1–2	1.474 (1.344 – 1.617)	<0.001
	3 or more	2.442 (2.220 – 2.687)	<0.001
	Length of stay		
	0 – 1 days	1.349 (1.228 – 1.482)	<0.001
	2 – 9 days	Reference	
	10 or more days	1.149 (1.051 – 1.257)	0.002
	**Clinical variable**	**Ods Ratio (95**% **CI)**	**p value**
Linear regression for length of stay (n = 34005)	Constant	2.590 (2.077 – 3.103)	<0.000
	Provider Group		
	Traditional-FP	Reference	
	FP-Hospitalist	0.096 (−0.136 – 0.329)	0.417
	GIM-Hospitalist	−2.975 (−3.302 – -2.647)	<0.001
	Other IM	−3.592 (−3.829 - -3.355)	<0.001
	Charlson score		
	0	Reference	
	1–2	0.672 (0.512 – 0.832)	<0.001
	3 or more	1.986 (1.816 – 2.157)	<0.001
	Age in years	0.058 (0.054 – 0.061)	<0.001
	Transfer in from other acute care hospital	0.901 (0.570 – 1.232)	<0.001
	Number of interventions	1.997 (1.937 – 2.056)	<0.001
	Emergent admit	−0.583 (−0.996 - -0.170)	0.006

Legend: FP = family physician, GIM = general internal medicine, IM = internal medicine, CI = confidence interval.

**Table 5:** Hospital Standardized Mortality Ratios (HSMR) for various physician provider groups

**Provider group**	**HSMR (95% confidence interval)**
Traditional-FP	108.52 (94.94 – 123.49)
FP-Hospitalist	105.18 (99.48 – 111.12)
GIM-Hospitalist	61.22 (48.97 – 75.61)
Other IM	99.34 (92.01 – 107.12)

Legend: FP = family physician, GIM = general internal medicine, IM = internal medicine.

We repeated our analysis after combining GIM and FP-Hospitalists into a single group (results not shown). Our results were similar to earlier comparisons, demonstrating improved mortality, readmission and LOS compared to traditional-FPs, and similar performance to other-IM with the exception of LOS where other-IM performed better.

## Discussion

Despite the widespread adoption of hospitalist programs in Canada, little evidence about their effectiveness exists. Previous studies have methodological limitations [8] and are mostly confined to reporting unadjusted before and after measurements [12,13].

We find that in our institution, both FP-Hospitalists and GIM-Hospitalists appear to perform better on the main outcome measure of hospital mortality compared to traditional FPs. These differences seem to persist if HSMRs are compared using independently derived methodologies developed by CIHI. Previous studies from the U.S. have largely failed to show a mortality benefit for hospitalist programs, but such evidence may not be directly applicable to Canada. Compared to subspecialists, the magnitude of potential mortality benefit with hospitalists in our institution does not appear to be as consistent, ranging from worse outcome with FP-Hospitalists in logistic regression to better outcome with GIM-Hospitalists in HSMRs.

The reduction in 30-day readmission rates realized by our hospitalist programs compared to traditional FPs is also of note, especially as payers focus on system efficiencies in response to economic pressures. Moreover, this finding suggests that some of the inherent discontinuity of care introduced by hospitalists may be offset by other benefits such as 24-hour availability of physicians and better familiarity with health system processes.

Our study shows that the effect of hospitalists on resource utilization (as represented by LOS) is more mixed. Compared to traditional FPs, GIM-Hospitalist patients have a statistically significant lower LOS (p < 0.001). They also appear to perform similarly to other-IM. On the other hand, FP-Hospitalists demonstrate a similar or worse LOS compared to traditional provider groups. Previous studies have suggested that fragmentation of care due to a high number of hospitalists rotating through a patient’s hospitalization episode increases LOS [14]. With 16 full-time equivalent physicians, the FP-Hospitalist program is the largest in our hospital and thus possibly exhibits the most discontinuous care. A prior internal audit revealed that in 2007–2008, an average of 2.46 FP-Hospitalists were involved per medical case (data not shown). In our study we were unable to account for the potential impact of the number of providers on LOS due to limitations of CIHI DAD data set. Nonetheless, our findings suggest that FP-Hospitalists can have at least an equal resource utilization compared to traditional FPs, despite caring for older and sicker patients.

An unexpected finding in our study is the degree of differences observed between FP- and GIM-Hospitalist groups, given broad similarities in patient populations and intertwined care processes. This finding may partly be due to the inherent limitations of using administrative databases and statistical adjustment. Additionally, the work distribution of the two groups is such that FP-Hospitalists tend to have a higher proportion of palliative patients, and also look after more oncology and nephrology patients. However, the possible contribution of training background cannot be discounted. Post-graduate family medicine training in Canada is primarily focused on outpatient care and is considerably shorter in duration than general internal medicine training which is mostly hospital-based. Even in the United States, where family medicine and internal medicine are more similar in terms of length of training and a shared focus on primary care, differences in communication skills, diagnostic certainty and resource utilization have been described [15-18] suggesting potential differences in core competencies and philosophy of care.

Our study has a number of notable features. To our knowledge, our study is the first attempt to systematically evaluate the quality of care delivered by Canadian hospitalists compared to traditional care providers. As well, our study attempts to overcome limitations of other studies by controlling for confounding factors. The hospitalist programs at LH have been in operation for many years, and are amongst some of the more mature programs in Canada. We have previously described a “maturity curve” for Canadian hospital medicine programs [19]. Both programs at LH can be considered “third generation” programs with significant levels of involvement in institutional processes. This “stability” allows for a meaningful assessment of program performance. Similarly, the availability of two hospitalist programs staffed by physicians of different training backgrounds is unusual and provides a unique opportunity for a comparative assessment.

Our study has a number of limitations. First, we retrospectively used an administrative database to compare the performance of different care models that comprise a complex array of processes. While we have made efforts to identify and adjust for confounding factors, our analysis may be limited by the lack of important data points in the Discharge Abstract Database For example, the DAD does not include information on adverse events that could impact hospital mortality and readmission rates. As well, the adjusted r^2^ of the LOS linear regression is quite low, indicating that there are likely many other variables affecting this length of stay not captured in our dataset. Second, we were not able to obtain costing analysis to compare efficiencies between the various study groups. Third, we have studied programs at a single institution in Ontario, and our results may not be applicable to other settings. For example, in a recent abstract from an academic institution in the same province, no statistically significant benefit to an internist-based hospitalist program was found when compared to Clinical Teaching Units staffed by academic general internists [20]. Finally, the geographic assignment of the two hospitalist groups may have an impact on outcome measures through differences in case mix and implementation of patient safety and quality improvement projects on different units at different times. For example FP-Hospitalists are responsible for patient care on the main oncology-nephrology ward, as well as the rehab units while the GIM-Hospitalist group has a higher proportion of cardiology patients.

## Conclusions

Our findings suggest that compared to a traditional FP model, and possibly the traditional subspecialist model, hospitalist-based programs might improve quality of care through reductions in in-hospital mortality and 30-day hospital readmissions. Moreover, hospitalists might improve system efficiencies by reducing LOS. Hospitalist training background does appear to influence the magnitude of potential benefit, a finding which may have implications for developing national hospital medicine core competencies and training programs. A formal randomized trial of hospitalist programs is unlikely to occur, and thus further research in this area is likely to remain grounded in observational studies. As such, a fuller body of literature is needed prior to drawing firmer general conclusions on the effectiveness of hospitalists and the impact of training background.

## Appendix A: Details of methodology

### Exclusion criteria

– Surgical case defined as Therapeutic Intervention CCI code with 4th and 5th digit equal to 50 or greater (1.XX.50.XX) or Obstetrical/Foetal Intervention CCI code with 4th and 5th digit equal to 45 or greater (5.XX.45.XX). These are the same definitions used by CIHI when calculating national HSMR data.

– Psychiatric cases with Most Responsible Discharge Diagnosis ICD 10-A codes of F20-F44 and F46-F98. Codes F10-F19 and F45 are included because these include various dementias, drug withdrawals and somatisation which hospitalists will typically admit instead of psychiatrists.

– Obstetrical cases with Most Responsible Discharge Diagnosis ICD 10-A codes of O00-O08, O22-27, O29, O30-O84, O86, O89, O91, O94-O97. Cases with other obstetrical codes are included because they include possible medical-related pregnancy conditions that hospitalists may treat (e.g. pregnancy-related venous thromboembolism or sepsis).

– Age < 18 years

– Main provider service is non-medical program

### Multivariate binary logistic regression (for mortality and readmission)

Binary logistic regression was used to calculate adjusted odds ratios for hospital mortality and 30-day readmission; for the readmission regression, the model excluded deaths as patients cannot be readmitted upon dying. All readmissions were considered independent encounters. Age, gender, transfer status, length of stay, elective or urgent category, Charlson score, number of interventions, discharge destination and number of diagnoses were first examined in univariate analysis to select candidate predictors. Significant variables were then entered as covariates using the backward Wald procedure in SPSS. Variables were first entered as continuous if possible, and then examined as categorical. Physician provider group and age were entered as a mandatory variable. Nagelkerke R2 and Hosmer-Lemeshow statistics were used to evaluate model fit. Nearing the construction of the final model, covariance matrices for correlations of estimates were performed to check for co-linearity. Variables with collinear relationships were kept if the standard error of the beta coefficient was small and retaining the variables improved model fit. AUROC was performed to test for model discrimination. For the hospital mortality regression, the AUROC was 0.767. For the readmission regression, the AUROC was 0.620.

### Multivariate linear regression (for length of stay)

To assess predictors of LOS, we assessed age, gender, transfer status, length of stay, elective or urgent category, Charlson score, number of interventions, and number of diagnoses for significance in univariate analysis. Variables that were significant at p < 0.10 were then entered as covariates in a forward step-wise general linear regression model. Variables that continued to be significant at a two-sided level of p < 0.10 were kept as main effects. A tolerance measure of 0.6 was accepted for co-linearity. Provider group was forced into the model and was exempted from the significance and tolerance criteria. The Durbin-Watson statistic was used to assess for independence. Plots of standardized residuals were examined to identify outliers; based on this analysis, we only included length of stay days that were 40 days or less. Plots were examined to check the normality assumptions of the residues. The adjusted R^2^ of the model was 0.249.

## Competing interests

VY and CC are both hospitalists at LH. Apart from providing the raw data to the investigators, Lakeridge Health has not had any involvement in the design of the study or publication of this paper. The investigators have not received any funding from any sources including LH. VY is the co-founder and CEO of Hospitalist Consulting Solutions, a healthcare consulting company that specializes in helping organizations design and implement hospital medicine programs in Canada.

## Authors’ contributions

VY conceived of the study and obtained the raw data. CC preformed the statistical analysis. Both authors participated in the writing of the manuscript. All authors read and approved the manuscript.

## Pre-publication history

The pre-publication history for this paper can be accessed here:

http://www.biomedcentral.com/1472-6963/13/204/prepub
